# Distribution of Antibiotic Resistance Genes in Three Different Natural Water Bodies-A Lake, River and Sea

**DOI:** 10.3390/ijerph17020552

**Published:** 2020-01-15

**Authors:** Sicong Su, Chenyu Li, Jiping Yang, Qunying Xu, Zhigang Qiu, Bin Xue, Shang Wang, Chen Zhao, Zhonghai Xiao, Jingfeng Wang, Zhiqiang Shen

**Affiliations:** 1 Department of Environment and Health, Tianjin Institude of Environmental and Operational Medicine, Tianjin 300050, China; 2 Heping District Center for Disease Control and Prevention, Tianjin 300020, China; 3 School of Public Health, Nanchang University, Nanchang 330006, Jiangxi, China

**Keywords:** water body, ARGs, absolute abundance, redundancy analysis

## Abstract

Currently, due to abuse in the use of human antibiotics and the weak regulatory control that the authorities have over sewage discharge and manure management, antibiotic resistance genes (ARGs) have become a new type of environmental pollutant. Three different natural water bodies (Poyang Lake, Haihe River and Qingdao No.1 Bathing Beach seawater) were sampled during the same periods to conduct a longitudinal comparison of distribution. The distribution and expression of 11 ARGs in 20 species were studied, and the correlations between the expression and the distribution of time and space of the ARGs in different water bodies were also analyzed. With the exception of *ermA*, *blaNDM-1* and *vanA*, which were not detected in seawater, the other ARGs could be detected in all three water bodies. Tetracycline resistance genes (*tetC*, *tetM* and *tetQ*) in the seawater and Haihe River had even reached 100%, and sulfa ARGs (*sul1* and *sul2*) in the seawater and Poyang Lake, as well as *sul2* and *sul3* in the Haihe River, had also reached 100%. The ARG pollution in Haihe River was much more serious, since 14 and 17 of 20 ARG species were significantly higher compared with seawater and Poyang Lake, respectively. Some ARGs also had a high absolute abundance. The absolute abundance of macrolide resistance genes (*ermB*) in seawater was as high as 8.61 × 10^7^ copies/L, and the anti-tuberculosis resistant genes (*rpoB* and *katG*) in the Haihe River Basin were highly abundant at 1.32 × 10^6^ copies/L and 1.06 × 10^7^ copies/L, respectively. This indicates that ARGs have gradually become more diverse and extensive in natural water bodies. The results of a redundancy analysis (RDA) of the three water bodies showed that although each water body is affected by different factors in space and time, overall, the presence of AGRs is closely related to the production and life of human beings and the migration of animals.

## 1. Introduction

The problem of antibiotic resistance has increasingly become one that plagues countries around the world. Antibiotics usually play an irreplaceable role in the treatment of infectious diseases, and they are used not only in human medicine but also in veterinary practices, animal husbandry, agriculture and aquaculture [1]. The mass of antibiotics discharged into the surface water, groundwater and ocean eventually leads to the rapid propagation of antibiotic resistance genes (ARGs). Past research has primarily suggested that high levels of clinically relevant ARGs come from selection pressure caused by antibiotic pollution [2], but the latest research shows that the existence of resistance genes can be largely explained by fecal contamination, not the selective pressure previously thought [3]. From the late 1960s to the early 1980s, the pharmaceutical industry tried to solve the problem of antibiotic resistance by continuously developing new antibiotics, which also contributed to the current worldwide phenomena of antibiotic abuse. Thereafter, the research on antibiotics began to slow down, and the number of new antibiotics has continuously decreased [4]. However, the threat of antibiotics to humans has not diminished, and according to one study, the number of deaths due to antibiotic resistance may reach 10 million annually by 2050 unless humans fail to take appropriate measures [5]. Humans are still threatened by bacterial infections. MRSA [6], VRE [7], epidemics of *Streptococcus pneumoniae* and *Mycobacterium tuberculosis* [8] and the emergence of multi-drug resistant (MDR) Gram-negative bacilli [9] have already seriously harmed human health. Currently, ARGs as contaminants of continuous concern, due to their role in the spread of antimicrobial resistance, have been currently recognized as being present in the environment [10]. In related research on ARGs, their transport and distribution are a hot issue in environmental science [11]. For example, the use of fertilizers in farmland is a potential route for the transmission of antibiotic-resistant bacteria from livestock to crops, animals and humans [12]. Rainfall will have a great impact on the abundance of the regional ARG [13]. In addition, it has been reported that microorganisms are specific at a high taxonomic level, which is closely related to the spread of ARGs. For example, distinct *int1* genotypes are induced for the expression of the *sul1* and *ermF* genes in Gammaproteobacteria and Bacteroidetes [14]. Among the factors that affect the transport and distribution of ARGs, water is one of the most important factors. Not only can ARGs diffuse because of the fluidity of water bodies they can also be transferred between bacteria via horizontal gene transfer leading to their wide spread.

Water bodies are major reservoirs of ARGs due to their long-term absorption of various pollutants. Therefore, it is of substantial importance to investigate the distribution, abundance and variation of ARGs in natural waters for water quality assessment and pollution control. In recent years, numerous types of research have been conducted to investigate the distribution and diversity of ARGs in different bodies of water, such as urban lake surface water [15], drinking water [16], coastal water [17] and tap water [18]. However, these studies of ARGs in water tend to be conducted in the same season although some seasonal sensitive factors such as water flow, human activity and bird migration may influence the abundance of ARGs. Even if there are occasional studies involving seasonal factors [19,20,21], such research often studies the influences of seasonal factors on a single water body and lacks the horizontal comparison of time factors between different water bodies, which can demonstrate the seasonal stability difference of abundance and distribution of ARGs between water bodies. Therefore, to study the ARGs, which are abundant in lakes, rivers and seas, our team spent approximately half a year sampling three representative natural water bodies—Poyang Lake, Haihe River and Yellow Sea during high and low water periods to investigate: (1) current distribution of ARGs in typical bodies of water. (2) Differences in the expression of ARGs in different bodies of water during the same period. (3) Impacts of different time and geographic locations on ARGs.

## 2. Materials and Methods

### 2.1. Sampling Sites and Time

**Figure 1:**
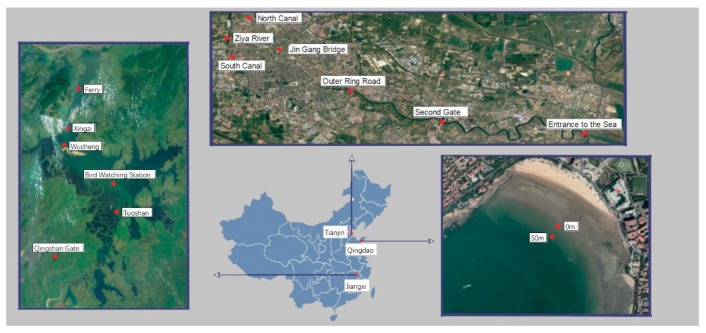
Schematic diagram of sampling locations in Poyang Lake, Haihe River and Qingdao No.1 Bathing Beach.

This study set sampling sites at three representative natural water bodies: Poyang Lake—China’s largest freshwater lake, Haihe River—one of the largest river in northern China and Qingdao No.1 Bathing Beach (Yellow Sea)—a typical offshore seawater (Figure 1). More specifically, six representative sampling points were selected from Poyang Lake, the principles of which were determined as follows: Qingshan Gate was selected to investigate the impact of various urban sewage; Tuoshan and Bird Watching Station were selected to consider the influence of bird migrations, and Wucheng, Xinzi and Ferry were selected to assess the influence of human activities. From Haihe River, the sampling points included three upstream sites (North Canal, Ziya River and South Canal), three midstream sites (Jin Gang Bridge, Outer Ring Road and Second Gate) and one downstream site (entrance to the sea). In Qingdao No.1 Bathing Beach, the sampling points of seawater were selected at 0 m and 50 m offshore. The sampling time of the lake and river was selected in May and June of the flood seasons and in November and December of the dry season. The sampling time of the seawater was kept consistent with the samples described above. The time spanned from May to December 2017. The volume of water sampled was 2 L from each sampling point, and the samples were stored in pre-sterilized polyethylene plastic drums, transported in the dark and treated within 4 h.

### 2.2. DNA Extraction, Conventional PCR and qPCR

#### 2.2.1. DNA Extraction

Each 1 L water sample was collected in triplicate at selected sites and filtered through a 0.22 μm polyethersulfone (PES) micropore filter (Millipore, Temecula, CA, USA). The filtered membranes were placed in 30 mL normal saline and then eluted in a shaker with a temperature of 37 °C and a speed of 200 rpm for 2 h. The eluents were stored at −20 °C for later use. The DNA in the eluents was extracted using a bacterial genomic DNA extraction kit (TIANGEN, Beijing, China), suspended in a final volume of 50 μL and further purified using an Agarose Gel DNA Recovery Kit (TIANGEN) to minimize inhibition of the PCR.

#### 2.2.2. Conventional PCR

In this study, 20 ARGs in 11 antibiotics were selected as the study subjects. Seven of them (ß-lactam, macrolide, sulfonamide, tetracycline, aminoglycoside, chloramphenicol and vancomycin resistant genes) have been frequently reported to be detectable in water [1]. In addition, we selected resistance genes for rifamycin and isoniazid, which are commonly used in the treatment of tuberculosis, and resistance genes for the broad-spectrum antibiotics quinolone and trimethoprim used in clinical practice. Conventional PCR analysis of the ARGs was conducted in a volume of 20 μL using Bio-Rad MyCycler PCR equipment (Bio-Rad, Hercules, CA, USA) according to the manufacturer’s instructions. Detailed information on the ARG primers tested in this study (*tetB*, *tetc*, *tetM*, *tetQ*, *ermA*, *ermB*, *sul1*, *sul2*, *sul3*, *blaNDM-1*, *ampC*, *blaTEM*, *qnrA*, *aadA*, *aph(2′)-Id*, *catA*, *rpo B*, *dfrA1*, *vanA* and *katG)*, annealing temperature, amplicon length and corresponding references are listed in Supplementary Table S1.

#### 2.2.3. qPCR

Standards for the qPCR assays were prepared from the genomic DNA of *E. coli* DH5a (Takara Bioengineering Co., Ltd., Dalian, China). Seven point standard curves were generated by a 10-fold serial dilution of plasmids carrying 10^8^–10^2^ copy number of the target genes, and qPCR was performed using SYBR Green Master (Roche Diagnostics GmbH, Cambridge, MA, USA) and an ViiA™7 Dx Sequence Detection System (Applied Biosystems, Foster City, CA, USA) with cycling conditions of 95 °C for 10 min, followed by 40 cycles of 30 s at 95 °C and 60 s at 60 °C. All standard curves had R2 values above 0.99.

### 2.3. Statistical Analysis

All statistical analyses were performed using SPAW Statistics 18 software (SPSS, Inc., Chicago, IL, USA). The data was tested by an analysis of variance (ANOVA) and least significant difference (LSD).

The correlations within time, geographic locations and ARGs in three different water bodies were examined using redundancy analysis (RDA), which was conducted using R software (version 3.5.2) with “Vegan” packages and Canoco (version 4.5) software. 

## 3. Results

### 3.1. ARGs Occurrence in Water Bodies

**Figure 2:**
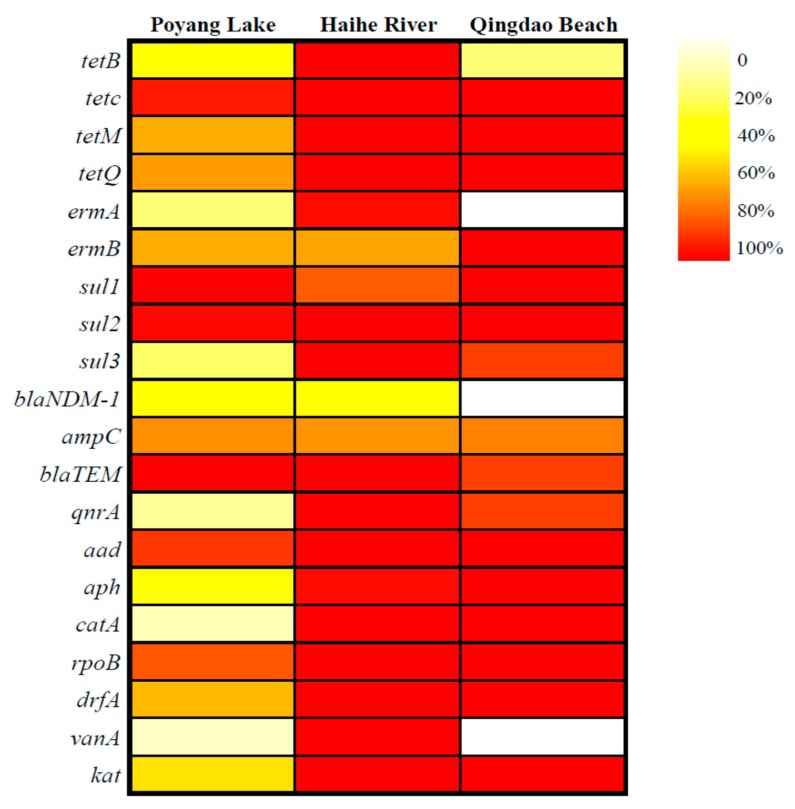
Heatmap of the detection rate of antibiotic resistant genes (ARGs) in three water bodies. Red indicates a higher detection rate, while white indicates a lower one.

The rate of detection of 20 species of 11 resistance genes was converted into a heatmap as shown in Figure 2. With the exception of *ermA*, *blaNDM-1* and *vanA*, which were not detected in seawater (Qingdao No.1 Bathing Beach), all the other ARGs could be detected in three water bodies. The tetracycline resistance genes (*tetB*, *tetC*, *tetM* and *tetQ*) had a high rate of detection in all three water bodies. An exception was that of *tetB* in seawater, which was 37.50%. The other samples were all higher than 50%, and those of *tetC*, *tetM* and *tetQ* in seawater and the Haihe River even reached 100%. Furthermore, the rate of detection of sulfa ARGs (*sul1*, *sul2* and *sul3*) was similar to that of the tetracycline resistant genes. With the exception of the rate of detection of *sul3* in seawater, all the other samples were higher than 50%, while the rate of detection of *sul1* and *sul2* in seawater and Poyang Lake, as well as *sul2* and *sul3* in Haihe River, reached 100%. The overall rate of detection of the macrolide resistance gene *ermB* was higher than that of *ermA*, and the rate of detection of *ermB* in seawater was 100%. The rate of detection of the β-lactam resistance gene *blaTEM* was higher than that of *blaNDM-1* and *ampC*, which was 100%, 95.83% and 87.50% separately in the river, lake and sea, respectively. The aminoglycoside resistance genes *aad* and *aph* also showed a high rate of detection, and their rate in the seawater and *aad* in Haihe River both reached 100%. The rate of detection of other resistance genes (*qnrA*, *catA*, *rpoB1*, *drfA1*, *vanA* and *katG*) in the Haihe River all reached 100%. On average, the rate of detection in Poyang Lake was the lowest between the other two, and the Haihe River was the highest.

### 3.2. RT-qPCR Analysis of ARGs

**Figure 3:**
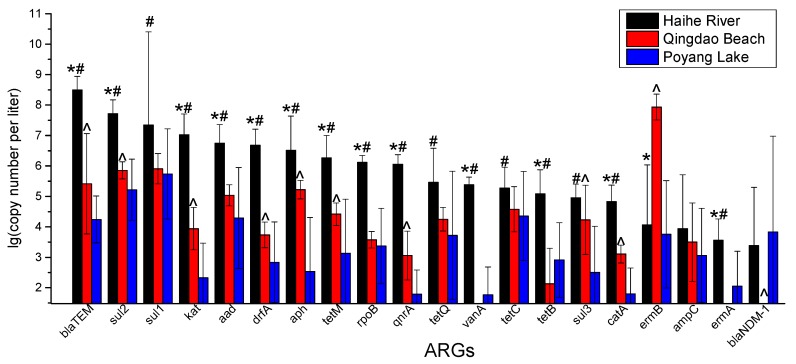
Absolute abundance of 20 ARGs in three water bodies. ‘*’ indicates *p* < 0.05 between Haihe River and Qingdao Beach; ‘#’ indicates *p* < 0.05 between Haihe River and Poyang Lake, and ‘^’ indicates *p* < 0.05 between Qingdao Beach and Poyang Lake.

The absolute abundance of ARGs in three waters is shown in Figure 3. In general, the absolute abundance of ARGs in the Haihe River was much higher than that in the other water bodies. Fourteen of 20 ARGs in Haihe River were significantly higher than those in the seawater (*p* < 0.05), while 17 of 20 ARGs in Haihe River were significantly higher than those in Poyang Lake (*p* < 0.05). Alternatively, the absolute abundance of ARGs in seawater was a little higher than in Poyang Lake. 11 of 20 ARGs in the seawater were significantly higher than in Poyang Lake (*p* < 0.05). More specifically, among the sulfa ARGs, the absolute abundance of *sul1* and *sul2* was significantly higher than that of *sul3* (*p* < 0.05), and the absolute abundance of *sul2* in the Haihe River was as high as 5.23 × 10^7^ copies/L. Among the tetracycline resistance genes, the absolute abundance of *tetC* in seawater and Poyang Lake was higher than that of *tetB*, *tetM* and *tetQ*, and the absolute abundance of *tetM* and *tetQ* in the Haihe River was one to two orders of magnitude higher than the other two, which were 1.83 × 10^6^ copies/L and 2.89 × 10^5^ copies/L. In all three water bodies, the absolute abundance of the macrolide resistance gene *ermB* was significantly higher than that of *ermA* (*p* < 0.05), and the absolute abundance of *ermB* in seawater was as high as 8.61 × 10^7^ copies/L. Among the *β-lactam* resistance genes, the absolute abundance of *blaTEM* was the highest in all three water bodies. Among the anti-tuberculosis resistant genes, the absolute abundance of *katG* in seawater and Haihe River was the highest, while *rpoB* was the highest in Poyang Lake, and the absolute abundance of *katG* in Haihe River was as high as 1.06 × 10^7^ copies/L.

### 3.3. Redundancy Analysis of the Correlations

**Figure 4:**
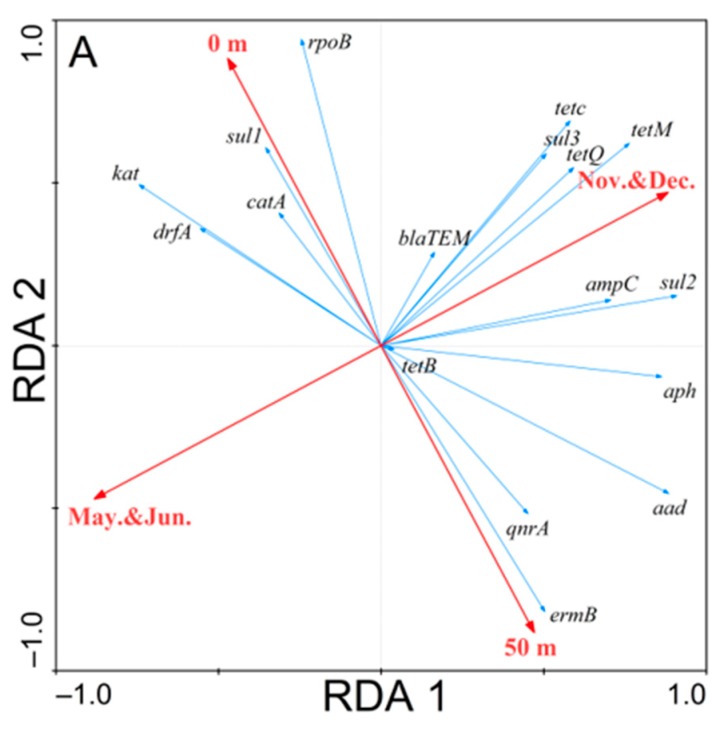

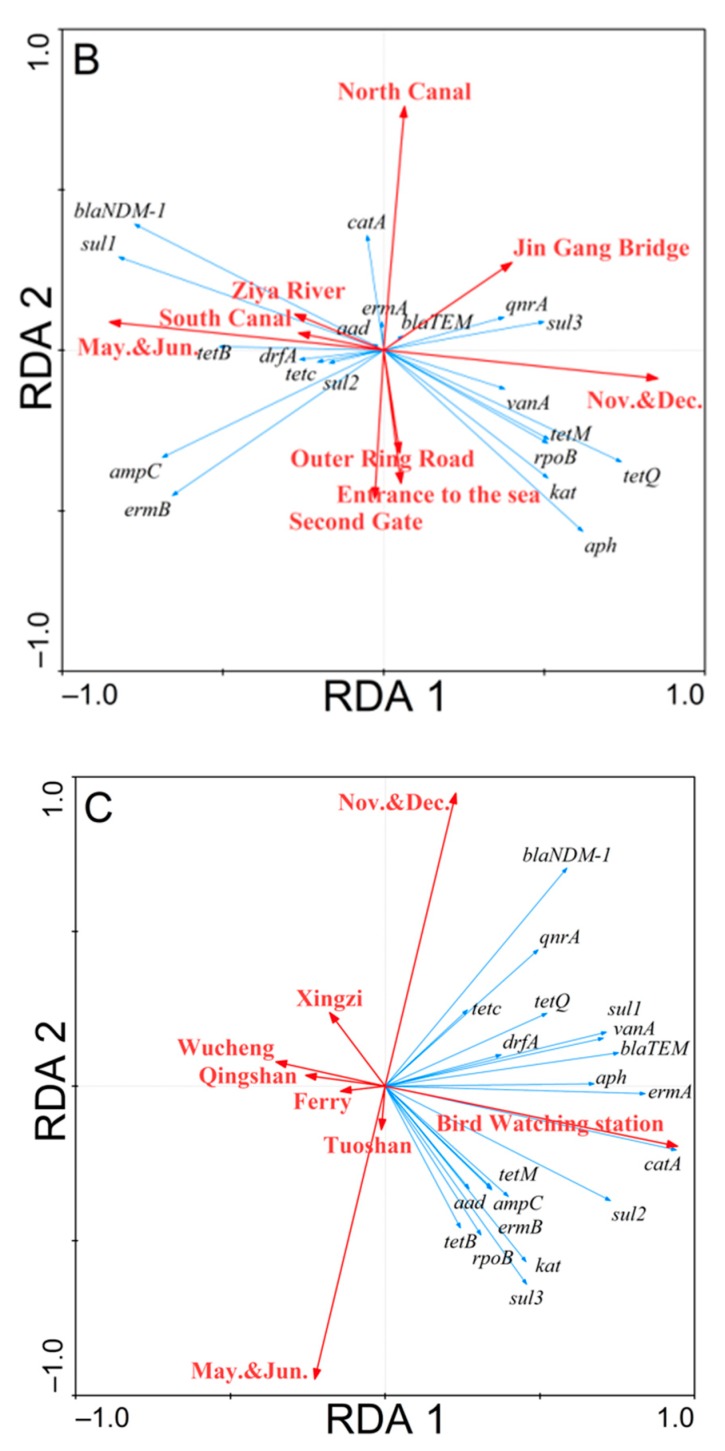
Redundancy analysis of three water bodies: (**A**) Qingdao No.1 Bathing Beach seawater; (**B**) Haihe River and (**C**) Poyang Lake.

A redundancy analysis of three water bodies showed that there were multiple correlations between the expression of different ARGs with time and spatial positions (Figure 4). In terms of time distribution, there were both 10 ARGs in the Poyang Lake body that showed temporal correlations with each two periods. In the spatial position, Tuoshan and Bird Watching Station showed significant correlations with all the ARGs tested. As could be seen from the analyses of the Haihe River basin, 11 and nine ARGs showed temporal correlations with two periods, respectively. In terms of spatial positions, it was found that the detection rates of ARGs in upstream waters with frequent population activities and midstream waters with prosperous poultry, livestock and fisheries were higher than those in downstream waters with industries and agriculture, but the differences were not significant. For seawater, 12 of the 17 ARGs detected showed a significant correlation in autumn and winter. In terms of spatial positions, nine and eight ARGs showed correlations between the two sampling sites, respectively.

## 4. Discussion

### 4.1. Detection of the ARGs

The ARGs were determined using conventional PCR analysis, and the results showed that the total rates of detection of ARGs in the three natural waters were at a high level. With the exception of *ermA*, *blaNDM-1* and *vanA*, which were not detected in seawater, all the other ARGs could be detected in three water bodies. The detection rate of ARGs was the highest in the Haihe River basin, which is more abundant and diverse than the other two water bodies. This may be related to the large amount of man-made pollution (particularly feces) discharged into the water, as the Haihe flows directly through the city. The related literature also reports that the spread and evolution of ARGs is likely caused by anthropogenic pollutants [22]. The rates of detection of ARGs in the Poyang Lake were relatively lower, which may be related to the government’s better ecological protection measures in Poyang Lake. Three sulfonamide resistance genes (*sul1*, *sul2* and *sul3*) and four tetracycline resistance genes (*tetB*, *tetC*, *tetM* and *tetQ*) were widely distributed in three water bodies. This may be due to the abuse of sulfonamides and tetracycline antibiotics in veterinary medicine and feed, which are excreted into the water along with animal feces, as well as the self-amplification of the resistance genes and their stability in the environment [23]. In addition, *qnrA*, *aadA*, *dfrA*, *rpoB* and *katG* were detected in all samples of seawater and river water, indicating that the ARGs in the Haihe River and the bathing beach were more diverse. The bacteria that cause tuberculosis often develop drug resistance, which can lead to prolonged disease progression in patients [24]. The rates of detection of *rpoB* in three water bodies were as high as 100%, 100% and 75%, indicating that *rpoB* has been widespread in various waters. It is urgent to take more care to prevent the further spread of anti-tuberculosis drug resistance genes in the environment.

### 4.2. Absolute Abundance of ARGs

To a certain extent, the absolute differences in abundance of the ARGs can reflect the degree of their pollution to different regions in different seasons. Several of the 11 classes of the 20 ARGs quantified in this study were highly expressed at each sampling point (Figure 3). The ARG pollution in Haihe River was much more serious, because it is a river flowing through Tianjin urban area (15.596 million permanent population, 384 people/km^2^ average population density of Haihe River Basin), and the city has absolutely higher population densities and activities than the beach, which are only open for 3 months each year and Poyang Lake, which is a natural environmental protection area. This is most likely due to the manures excreted by humans, poultry, livestock and fish are discharged with wastewater into the river.

The sulfonamide resistance genes were primarily *sul1* and *sul2* genes. The *sul1* gene was also the most abundant of the 20 resistant genes tested. Currently, sulfa antibiotics are widely used in aquatic products and animal husbandry industries and discharged with feces, but they are not easily degraded in the environment [25], resulting in high levels of sulfonamide resistance gene residues. The *sul1* genes are located on large binding plasmids, and the *sul2* genes are located on widely distributed plasmids and small unbound plasmids in the host. Therefore, the high abundance and broad distribution of *sul1* and *sul2* may be owing to the binding plasmids [26]. Due to poor management of animal excrement discharge, the abundance of sulfa ARGs in China’s water environment are at a high level, and the Haihe River Basin is representative of this situation.

The tetracycline resistant genes *tetB*, *tetC*, *tetM* and *tetQ* were selected for this study. The major genotypes of tetracycline resistant genes in different water bodies were also different. In lake water and seawater, the absolute abundance of *tetC* was higher than that of the other four, while *tetM* was detected in river water. Different tetracycline resistance genes have varying resistance mechanisms. The resistance mechanisms produced by *tetB* and *tetC* are specific efflux pumps, which are mostly distributed in Gram-negative bacteria [27] The resistance mechanisms of *tetM* and *tetQ* are targeted modifications of ribosome protective proteins, which are mostly distributed in Gram-positive bacteria. The large proportion of Gram-negative bacteria in water may be one of the reasons for the high absolute abundance of *tetC*. Another study showed that *tetC* has a high rate of detection in lactose-fermenting Enterobacteriaceae isolated from activated sludge in sewage treatment plants (STPs) [28]. Since the sewage will enter the aquatic environment, it could indicate that the presence of tetracycline resistance genes in the water was caused by *Enterobacter* in the wastewater from STPs. This also suggested to some extent that the increase in *tetC* abundance might be related to fecal emissions.

Among the macrolide resistance genes, the absolute abundance of *ermB* was significantly higher than that of *ermA*, which was the main genotype in all water bodies. The macrolide resistance gene *erm* is easily transferred from one host to another via a plasmid or transposon [29]. Currently, a variety of *erm* genes (A, B, C, F, T and X) have been detected in poultry feed wastewater and livestock manure, and *ermB* is considered to be the most prevalent genotype in the environment [30]. Our study also found that *ermB* was more prevalent than *ermA*. Interestingly, the absolute abundance of *ermB* in seawater was much higher than that in the other two water bodies. The *ermB* is very common in human and animal microbiomes. From this, the important role of the marine environment in the development of antibiotic resistance and transmission of resistance genes between bacteria remains to be further elucidated [31].

Although aminoglycoside antibiotics have been restricted, the genes (*aadA* and *aph*) in this study still had reached certain abundance levels in all three water bodies. This suggested that ARGs could still be replicated and amplified by gene level transfer in the absence of selective antibiotic stress. The result also indicated that once the water environment becomes contaminated with ARGs, the ARGs will persist in the environment as pollutants that are persistent and difficult to eliminate.

β-lactam is the most widely used antibiotic in clinical treatments because of its low amount of side effects. It can be used in both human and veterinary medicine; therefore, a large amount of antibiotics have been released into the environment. The *blaNDM-1* gene is resistant to metallo-ß-lactamase and can degrade most antibiotics, including cephalosporins, penicillins, carbapenems and other ß-lactam antibiotics, leading to widespread antibiotic resistance [32]. The *blaTEM* gene is located on a mobile binding plasmid and transposon, enabling it to be transferred between different species. Bacteria carrying the gene *blaTEM* are resistant to penicillin. Due to the long history of human penicillin use, the *blaTEM* gene is often detected in the intestinal commensal coliforms of healthy adults [33]. The β-lactam antibiotics in the environment are sensitive to pH and temperature and easily degraded [34], which makes the *blaTEM* gene abundance in the environment lower than expected. In particular, the *blaNDM-1* resistance gene was not detected in all samples of seawater, which might be caused by ultraviolet radiation and high sea temperature during the day. The specific reasons merit further investigation.

Among the trimethoprim, rifampicin, isoniazid, quinolone and vancomycin resistance genes, the representative genes *dfrA1*, *rpoB*, *katG*, *qnrA* and *vanA*, respectively, were primarily detected. Trimethoprim antibiotics are generally not used alone, and they have a synergistic effect with sulfa drugs, which can increase the effect by dozens of times. Research by Zhang and Arabi indicated that both the detection and detection rates of the *dfrA1* gene in the environment were at a low level [35]. However, in our study, the absolute abundance of the *dfrA1* gene in the Haihe River was 4.79 × 10^6^ copies/L, which indicated that it had accumulated to a high level. Both *rpoB* and *katG* are anti-tuberculosis resistance genes. Point mutations in the *rpoB* gene result in resistance to rifampicin (RFP), and point mutations in the *katG* gene result in resistance to isoniazid (INH) [36]. Studies have shown that despite the overall decline in tuberculosis incidence, data-consistent simulations suggested that between 2013 and 2025, the incidence of multidrug-resistant tuberculosis (MDR-TB) might continue to rise after 2013 due to the overuse of clinical persistence medications [37]. The *rpoB* and *katG* genes in the Haihe River Basin had shown a high abundance of 1.32 × 10^6^ copies/L and 1.06 × 10^7^ copies/L, respectively. Clinically, vancomycin is used as the “last line drug” to treat serious infections when all other antibiotics are ineffective. In this study, the vancomycin resistance gene *vanA* was detected in both river water and lake water, but the absolute abundance was not high, and it was not detected in seawater. This finding still raises alarm about the spread of *vanA*.

### 4.3. Time and Spatial Distribution of ARGs

The distribution of ARGs was affected by many factors, including antibiotics, heavy metal ions, various types of sewage and rainfall. In terms of time distribution, with the exception of seawater, different ARGs had shown correlations with different time periods. In May and June, a large amount of rainfall brought pollution from the soil into the natural water bodies, which aggravated the pollution of the natural water bodies and increased the amount of ARGs [13]. However, a large amount of rainfall will also accelerate the river water flow and bring pollutants and ARGs into the downstream environment by the migration of water flow [38]. The ARGs in natural waters are highly susceptible to storm floods. Therefore, the abundance of ARGs in November and December can better reflect the pollution of natural water bodies. From the analytical results of seawater, it could be seen that 12 of the 17 ARGs detected have obvious correlations with autumn and winter. Since the beach is open to the public only after July of each year, there will be a large number of tourist activities on the shore at that time. Whether this is related to the influx of tourists after the beach opened and the route by which the ARGs enter the seawater merit further study. In addition, lower temperatures and weaker UV intensity in the autumn and winter might result in slower elucidation of some ARGs.

In terms of spatial positions, the Tuoshan and Bird Watching Station in Poyang Lake showed significant correlations with all the ARGs tested because Tuoshan and the Bird Watching Station are located in the center of the lake and belong to the bird nature reserve. Studies have reported that bird dung carries a large number of ARGs [39,40]. Therefore, migrations of migratory birds can carry the ARGs of the external environment to Poyang Lake. In addition, the ARGs of Qingshan Gate may also pollute the downstream water bodies under the migration of physical factors, such as rainfall, water flow and winds. The ARGs contamination of Tuoshan and Bird Watching Station may be caused by the migration of birds and upstream pollution. In addition, the long-distance migrations of migratory birds can also spread the ARGs in the Poyang Lake Basin to other parts of the world, eventually resulting in the global prevalent of ARGs. Therefore, the impacts of bird migrations on ARGs in the Poyang Lake basin are worthy of attention. It could be seen from the analysis of the Haihe River basin that the expression of ARGs was closely related to human activities. The North Canal, Ziya River and South Canal are located in densely populated areas of human lives, while Jingang Bridge, Outer Ring Road and Second Gate are located in poultry, livestock and fish farming areas. The ARGs detected at these sites are generally more abundant than those in other regions. This is most likely due to the manure excreted by humans, poultry, livestock and fish that are discharged with wastewater into the river.

## 5. Conclusions

Some of the common ARGs found in the human and animal microbiome can already be detected in natural water bodies, and the ARGs have been widely distributed in the natural environment through physical factors, such as rainfall, water flow, wind and animal migrations. In this study, we selected 15 sampling points for analysis in three different water bodies in China to study the distribution of ARGs. The results indicated that different types of ARGs were widely distributed in natural waters, such as the *sul*, *tet* and *erm* genes. In addition, the super-large Poyang Lake has already shown fairly abundant ARG pollution. Although the problems of ARG pollution have attracted some attention in recent years, there is still a need for more effort to reduce the possibility of ARGs entering and spreading into the environment.

## Supplementary Materials

The following materials are available online at https://www.mdpi.com/1660-4601/17/2/552/s1, Table S1. The primers used in this study.
