# Supplementary material for: Distribution of Antibiotic Resistance Genes in Three Different Natural Water Bodies-A Lake, River and Sea
Source: Int J Environ Res Public Health. 2020 Jan 15;17(2):552. doi: 10.3390/ijerph17020552 (PMC7014431; doi:10.3390/ijerph17020552)
Supplement: Supplementary file 1 [file ijerph-17-00552-s001.pdf]

Table **S1.** The primers used in this study.

Gene	Sequence (5'-3')	Annealing temperature (°C)	Amplicon Length	Reference
Bla_{NDM}	F-ATT AGC CGC TGC ATT GAT	60	154	[2]
	R-CAT GTC GAG ATA GGA AGT G			
ampC	F-AACAAAAGATCCCCGGTATGG	60	151	[3]
	R-ACGCCCGTAAATGTTTTGCT			
Bla_{TEM}	F-AGCATCTTACGGATGGCATGA	55	103	[2]
	R-TCCTCCGATCGTTGTCAGAAGT			
ermB	F-TAAAGGGCATTTAACGACGAAACT	60	172	[2]
	R-TTTATACCTCTGTTTGTTAGGGAATTGAA			
ermA	F-AAG CGG TAA ACC CCT CTG A	60	190	[2]
	R-TTC GCA AAT CCC TTC TCA AC			
sul1	F-CGCACCGGAAACATCGCTGCAC	55	163	[4]
	R-TGAAGTTCCGCCGCAAGGCTCG			
sul2	F-TCATCTGCCAAACTCGTCGTTA	55	105	[2]
	R-GTCAAAGAACGCCGCAATGT			
sul3	F-CCCATACCCGGATCAAGAATAA	58	143	[2]
	R-CAGCGAATTGGTGCAGCTACTA			

tetB	F-CGAAGTAGGGGTTGAGACGC	55	192	[2]
	R-AGACCAAGACCCGCTAATGAA			
tetC	F-GCGGGATATCGTCCATTCCG	55	207	[2]
	R-GCGTAGAGGATCCACAGGACG			
tetM	F-CATCATAGACACGCCAGGACATAT	60	101	[2]
	R-CGCCATCTTTTGCAGAAATCA			
tetQ	F-AGAATCTGCTGTTTGCCAGTG	58	124	[2]
	R-CGGAGTGTCAATGATATTGCA			
qnrA	F-AGGATTTCTCACGCCAGGATT	55	124	[1]
	R-CCGCTTTCAATGAAACTGCAA			
aadA	F-GTTGTGCACGACGACATCATT	55	102	[2]
	R-GGCTCGAAGATACCTGCAAGAA			
aph(2')-Id	F- TAAGGATATACCGACAGTTTTGGAAA	60	117	[2]
	R- TTTAATCCCTCTTCATACCAATCCAT			
catA	F-GGGTGAGTTTCACCAGTTTTGATT	55	101	[2]
	R-CACCTTGTCGCCTTGCGTATA			
vanA	F-AAAAGGCTCTGAAAACGCAGTTAT	55	150	[2]
	R-CGGCCGTTATCTTGTAAAAACAT			
dfrA1	F-GGAATGGCCCTGATATTCCA	60	95	[5]
	R-AGTCTTGCGTCCAACCAACAG			
rpo B	F-GGTCGCCGCGATCAAGGAGT	60	159	[6]

	R-GTGCACGTCGCGGACCTCCA			
katG	F-GAAACAGCGGCGCTGATCGT	55	209	[7]
	R-GTTGTCCCATTTCGTCGGGG			

[1] LI Liwen, ZOU Likou, ZHOU Yang, et al. Dynamics of bacterial diversity and antibiotic resistance of Escherichia coli in Fu River, Chengdu[J]. Journal of Lake Sciences, 2012, 24(1):96-103.

[2] Naas T, Ergani A, Carrër A, et al. Real-time PCR for detection of NDM-1 carbapenemase genes from spiked stool samples[J]. Antimicrobial agents and chemotherapy, 2011, 55(9): 4038-4043.

[3] Zhu Y G, Johnson T A, Su J Q, et al. Diverse and abundant antibiotic resistance genes in Chinese swine farms[J]. Proceedings of the National Academy of Sciences, 2013, 110(9): 3435-3440.

[4] Zhang A Y, Wang H N, Tian G B, et al. Phenotypic and genotypic characterisation of antimicrobial resistance in faecal bacteria from 30 Giant pandas[J]. International journal of antimicrobial agents, 2009, 33(5): 456-460.

[5] Zhang S, Lv L, Zhang Y, et al. Occurrence and variations of five classes of antibiotic resistance genes along the Jiulong River in southeast China[J]. Journal of Environmental Biology, 2013, 34(2 suppl): 345.

[6] Cavusoglu C, Karaca-Derici Y, Bilgic A. In-vitro activity of rifabutin against rifampicinresistant Mycobacterium tuberculosis isolates with known rpoB mutations[J]. Clinical microbiology and infection, 2004, 10(7): 662-665.

[7] Musser J M, Kapur V, Williams D L, et al. Characterization of the catalase-peroxidase gene (katG) and inhA locus in isoniazid-resistant and-susceptible strains of Mycobacterium tuberculosis by automated DNA sequencing: restricted array of mutations associated with drug resistance[J]. Journal of infectious Diseases, 1996, 173(1): 196-202.
